# Trends in the Psychometric Characteristics of NECO Mathematics Senior School Certificate Examination Over a Period of Five Years (2020-2024) among Osun State Candidates, Nigeria

**DOI:** 10.12688/f1000research.182391.2

**Published:** 2026-07-11

**Authors:** Adediwura Alaba Adeyemi, Babayemi Beatrice Oluwakemi, Odumbo Odunayo Ibukun

**Affiliations:** 1 Department of Educational Foundation, Faculty of Education, Obafemi Awolowo University, Ife-Ife, Osun state, 10222, Nigeria; 2 Department of Art and Social Science, Faculty of Art, Obafemi Awolowo University, Ife-Ife, Osun state, 10222, Nigeria; 3 Department of Humanities, Faculty of Education, Kampala International University - Western Campus, Bushenyi, Western Region, 00000, Uganda

**Keywords:** SSCE, NECO, Mathematics, Secondary Schools, Examination.

## Abstract

The study examined the psychometric characteristics of the National Examinations Council (NECO) Senior School Certificate Examination (SSCE) Mathematics test in Osun State, Nigeria, spanning from 2020 to 2024. A random sample comprising 10% of the total of 211,753 candidates was selected for the study. The examination item responses were used to examine three factors: item difficulty, item discrimination, and test reliability. The researchers used descriptive statistics, one-way ANOVA, and Scheffe post hoc tests to analyse the collected data. The results showed that item difficulty remained largely stable over the years, except in the most recent examination year, which exhibited a marked change. The five-year period showed major changes in item discrimination indices because item quality testing yielded different results, whereas overall item discrimination remained within acceptable limits. The KR-20 reliability coefficients were high throughout the study, indicating that the test maintained consistent internal consistency during the assessment. The study found that the NECO SSCE Mathematics examination is highly reliable but requires ongoing psychometric assessment to maintain standards across periods, including reliability, fairness, and validity.

**List of Abbreviations**
ANOVA: Analysis of Variance
BECE: Basic Education Certificate Examination
CTT: Classical Test Theory
Df: Degrees of Freedom
DIF: Differential Item Functioning
F: F-statistic (in ANOVA)
IRT: Item Response Theory
KR-20: Kuder–Richardson Formula 20
NECO: National Examinations Council
OECD: Organisation for Economic Co-operation and Development
OMR: Optical Mark Recognition

$\bar{p}$

: Mean Item Difficulty Index
rpbis: Point-Biserial Correlation Coefficient
SD: Standard Deviation
SSCE: Senior School Certificate Examination
Sig.: Significance (p-value)
WAEC: West African Examinations Council

## Introduction

Large-scale national public exams play a crucial strategic role in a country’s education system, particularly when certification, school transfers, or access to additional educational resources depend on exam outcomes. In Nigeria, the National Examinations Council (NECO) Senior School Certificate Examination (SSCE) Mathematics is considered a high-stakes assessment, used to obtain secondary school completion certificates, secure admission to tertiary institutions, and signal for the labour market. Consequently, the analysis of NECO Mathematics scores and the credibility of decision-making processes that depend on these scores across various such sessions are largely contingent on the examination’s psychometric quality. The characteristics of large-scale assessments that are common targets of evaluation are item difficulty, item discrimination, test reliability, and item bias from the psychometric perspective. Item difficulty refers to the proportion of candidates who can provide correct answers to an item. In contrast, item discrimination refers to the extent to which an item can distinguish between individuals of high and low ability. Reliability assesses the consistency of scores, whereas bias analysis examines whether differentially functioning test items are equivalent across subgroups (e.g., gender, school type, or location). All these indices collectively provide empirical evidence for the validity, fairness, and technical soundness of any examination. ^1, 2^

There has been growing concern among educational stakeholders in Nigeria over the past decade, driven by inconsistent student performance in public examinations, particularly in Mathematics. These inconsistencies could indicate disparities in instructional quality, curriculum coverage, or learner readiness, but they could also be due to inconsistencies in item quality and in the test-construction process. Empirical studies conducted in the past decade have shown that public examination items in Nigeria sometimes exhibit disparities in difficulty distribution, weak discrimination parameters, and occasional DIF, making scores from one year to the next incomparable. ^3, 4^

In contemporary research, trend analysis plays a vital role in the measurement literature, rather than merely single-year statistical analyses. The psychometric characteristics of examinations may change over time and may be necessary for assessment purposes. In the last decade, this has involved determining whether all essential characteristics of an examination remain fixed or exhibit systematic drift in difficulty, reliability, or bias. This also contains substantive discussions of high-stakes examinations such as NECO SSCE, which underscore the importance of hours in nurturing public trust and shaping government actions, particularly in international educational achievement comparisons. ^5, 6^

In Osun State, where Mathematics performance has remained a key policy concern, a systematic examination of psychometric trends provides valuable evidence for educational planning, test-development reforms, and accountability. Understanding how item characteristics have evolved from 2020 to 2024 can inform NECO’s item-writing practices, guide teacher preparation strategies, and support policymakers in interpreting examination outcomes more cautiously. Consequently, this study investigates trends in the psychometric characteristics of NECO SSCE Mathematics over five years, with a focus on candidates in Osun State, Nigeria.

The theoretical attributes of test items and test forms that provide empirically supported evidence of the quality, credibility, and fairness of measurement in educational assessments are the psychometric qualities. Fundamental to large-scale public examinations, as well as to ensuring that test scores adequately and reliably reflect the true abilities of examinees in the area of focus, is the evaluation of psychometric properties. This evaluation is a key component in the introduction, quality control, and scoring of high-stakes examinations such as national benchmark exams. One of the most widely studied psychometric indices is item difficulty, which measures the proportion of examinees who answer a given item or question correctly. A difficulty index can help explore whether test items are well aligned with the examination population and curriculum expectations. An item that is too easy or too difficult hardly contributes to good metric measurement and may distort score distributions and undermine test validity. A well-constructed public examination generally contains items applied in three different levels of difficulty-easy, moderate, and difficult- to ensure an optimal precision of measurement across the ability continuum. ^7, 8^ Item discrimination, closely related to question difficulty, is the extent to which an item differentiates between examinees with high and low background performance. Discrimination indices indicate the measurement quality of an item with respect to its reliability and overall test validity. When the discrimination coefficient for a given item is high, it provides a strong signal to the rank ordering of candidates around an ability point. However, low-discriminative items may introduce waiving-along noise and may also prevent an item’s associated ability level from being inferred. ^9, 10^ Reliability is another crucial psychometric property that denotes the consistency and stability of test scores across items, forms, or administrations. In the case of public examinations, internal consistency indices, like KR-20 or Cronbach’s alpha, are widely applied to estimate the degree to which conversation among the various test items about the target construct can be. According to, ^11, 12^ adequate reliability is a prerequisite for valid interpretation of scores, particularly for consequential decisions, such as certification and admission, that attach social consequences to an individual’s performance.

Over the past 10 years, studies have indicated that the psychometric properties of public examinations in Nigeria vary from year to year. Previous studies have analysed the NECO and WAEC Mathematics exams, noting that although the overall difficulty levels are sometimes similar across the two organizations, the discrimination indices vary significantly across test versions and years, indicating potential issues with item quality and calibration. ^13 14^ also reported similar findings in the content-specific analysis of the NECO examinations, with some items exhibiting weak discrimination and low psychometric capacity despite not being particularly difficult.

The current results highlight the importance of conducting regular psychometric evaluations and monitoring item parameters in public examinations. Tracking methodologies make it possible to detect fluctuations in psychometric quality; however, comparability of results across examinations collapses within-cohort examinations, since the general large-scale examination system that provides so much information would lose its credibility. Awareness and assessment of the psychometric characteristics over and across examination years, therefore, remains the key concern of any test producer, policy developer, or educational measurement specialist.

The National Examinations Council (NECO) was established as an alternative national examining body in Nigeria, tasked with conducting credible, valid, and reliable public examinations. Questions about the quality and comparability of NECO examinations, particularly in high-stakes subjects such as mathematics and the English language, have attracted sustained scholarly attention since their inception. As a result, a considerable body of empirical research seeks to ascertain and critique the psychometric characteristics of NECO test items under the frameworks afforded by Classical Test Theory (CTT) and Item Response Theory (IRT). Several studies conducted in the last decade have designed NECO examination items across subject areas, with a major focus on item difficulty, discrimination, dimensionality, and model–data fit. Using IRT-based approaches, researchers reported that some NECO multiple-choice test forms did not fully meet the unidimensional assumption and exhibited local item dependence and misfitting items in certain administrations. In fact, ^3^ in their psychometric study of the NECO English Language item, found discrepancies in item parameter estimates and instances of poor item fit, pointing to the weaknesses in item calibration and pretesting procedures and raising the urgency for continuous psychometric scrutiny on NECO examinations to ensure measurement precision and construct validity.

Empirical assessments in Mathematics have identified mixed psychometric relations across years. A comparison of Mathematics items from NECO and the West African Examination Council (WAEC) indicates that the two tests exhibit similar difficulty across administrations. Still, NECO Mathematics items, unlike WAEC Mathematics items, display higher within-test discrimination variability. Thus, the variability in the extent of discrimination would raise concerns about the uniformity of measurement standards and the stability of score interpretation over time. ^1^

Beyond item quality, research is increasingly focusing on fairness and bias in NECO examinations. ^15^ demonstrated, through their Differential Item Functioning (DIF) analyses, that some of the mathematics items in the NECO examinations were functioning differently across subgroups defined by gender, school type (public versus private), and location (urban versus rural), while controlling for candidates’ overall ability. This differential functioning poses a threat to score equating and may systematically favour or disadvantage particular individuals or groups, thereby invalidating any decision-making based on examination results. ^16, 17^

Research comparing differential item functioning (DIF) indices frequently links deviations from uni-dimensionality to the presence of item bias. For instance, when a math exam clearly assesses both mathematical reasoning and language skills simultaneously, or when other test-taking techniques are employed, item parameters become less predictable and differences among subgroups become more apparent. Thus, ^18, 6^ opined that the very idea of fairness becomes inchoate when the issues of test validity and measurement model are not carefully considered, and myriad empirical conditions, of which substantive-parameter tuning continues to mount, are not carefully considered.

The findings indicate that the NECO SSCE Mathematics examination constitutes a valuable component of Nigeria’s national assessment and certification system, although persistent psychometric challenges underscore the need for continuous quality assurance. Routine item analysis, longitudinal monitoring of item parameters, and systematic bias evaluation should be institutionalised to enhance the reliability and validity of examination outcomes, particularly in mathematics, where deficiencies in test quality may compromise the accurate assessment of students’ competence and influence educational decision-making.

Although this study did not employ formal test equating, its longitudinal analyses remain appropriate for evaluating trends in examination quality. The NECO SSCE Mathematics examinations are developed using consistent test specifications, content blueprints, and moderation procedures, providing a reasonable basis for cautious comparisons of item characteristics across administrations. Rather than assuming score equivalence between cohorts, the study focuses on patterns in item difficulty, discrimination, and distributional characteristics to identify trends, irregularities, and evidence of stability within the Classical Test Theory framework. The large and relatively comparable candidate populations further strengthen the robustness of these descriptive and inferential analyses. Accordingly, the observed variations should be interpreted as indicators of potential changes in test quality rather than definitive differences in examination difficulty or candidate performance. Future studies should incorporate formal test equating or Item Response Theory (IRT) models to improve comparability across examination years and strengthen longitudinal psychometric evaluation.

## Research objectives

The main goal of this study is to examine how the psychometric properties of Mathematics in the NECO SSCE have evolved from 2020 to 2024 for candidates in Osun State, utilizing Classical Test Theory as the analytical framework. The specific objectives of the study are to:

i. Evaluate trends in item difficulty indices of NECO SSCE Mathematics multiple-choice items from 2020 to 2024 among candidates in Osun State.
ii. Review the trends of item discrimination indices of NECO SSCE Mathematics items across these five examination terms.
iii. Evaluate the reliability of NECO SSCE Mathematics tests across the tests given in the five years.
iv. Compare yearly variations in psychometric characteristics (item difficulty, item discrimination and reliability) of NECO SSCE Mathematics examinations from 2020 to 2024.

### Research questions

i. What is the trend observed in the difficulty indices of NECO SSCE Mathematics multiple-choice items from 2020 to 2024 among Osun State candidates?
ii. How do the NECO SSCE Mathematics items’ discrimination indices vary across the five examination years (2020–2024)?
iii. What is the extent to which the reliability coefficients of the NECO SSCE Mathematics examinations remain consistent across the years 2020 to 2024?

### Hypotheses

i. The difference in item difficulty of NECO SSCE Mathematics examinations between 2020 and 2024 varies significantly.
ii. The item discrimination of NECO SSCE Mathematics examinations between 2020 and 2024 did not vary significantly.

### Methodology

The study employed a descriptive quantitative design, using NECO Mathematics examinations from 2020 to 2024 as the test data and student responses from mathematics candidates in Osun State schools. The study population comprised all individuals from Osun State who enrolled in and participated in the NECO SSCE Mathematics examination between 2020 and 2024. Data from the examination board indicate that 66,256 candidates registered in 2020, followed by 34,434 in 2021, 34,682 in 2022, 35,118 in 2023, and 41,263 in 2024, for a total of 211,753 candidates over the five years. These individuals came from public and private institutions and represented a range of ability levels and learning settings in the state of Osun. A representative sample needed for an in-depth psychometric evaluation was selected, taking into account the large population and the long-term aspect of the research. Proportional random sampling was performed, drawing a 10% portion of the total population; 21,175 individuals were sampled. The sampling method was designed to ensure that each test year was accurately represented in the study sample, in proportion to its prevalence in the overall population. As a result, the study maintained the population’s characteristics to facilitate comparison and enabled more robust trend comparisons. Using a proportional allocation, the target totals for each year are set at 6626 for 2020, 3443 for 2021, 3468 for 2022, 3512 for 2023, and 4126 for 2024. Randomly selecting samples from exam records in each year ensured that every candidate had an equal, independent probability of inclusion in the study. The primary instrument used for data collection in this study was an electronic spreadsheet of OMR data containing candidates’ item-level responses for the years 2020 to 2024. This study carefully and systematically analysed data from 2020 to 2024 to achieve the research goals and ensure a thorough evaluation of the psychometric properties of the NECO SSCE Mathematics examination. The Classical Test Theory (CTT) model was employed to provide robust evidence regarding the quality of the test items and overall assessment.

### Instrumentation

The instrument used in this study was the NECO SSCE Mathematics multiple-choice test items that are administered every year. Each test form had 60 items, which were scored as yes/no (1 = correct response, 0 = incorrect response). The questions covered the main areas of mathematics that align with the national curriculum. NECO headquarters.

### Data collection procedure

Secondary data, the scanned Optical Mark Record (OMR) for a period of five years under consideration, was collected from the NECO official database. The files included candidate responses at the item level for each year that was under investigation. Also, all datasets were anonymised to protect confidentiality and to meet ethical requirements throughout.

The initial phase of data cleaning and screening was essential to ensure that the dataset was complete, accurate, and suitable for the psychometric analysis. Since the study relied on secondary data from large exam response records, the screening was conducted tightly to reduce measurement error and improve the relevance of subsequent analyses. First, all response scripts on the OMR were reviewed for full coverage. Any scripts showing too many missing answers, operationally defined as more than 10% of the total items, were excluded from the dataset. This cut-off was used to avoid skewing item statistics, as higher levels of missingness can bias estimates of item difficulty and discrimination and possibly reduce reliability coefficients. The scoring accuracy for each item was also verified. Each response was rechecked to ensure the binary code was correct, with “1” for correct answers and “0” for incorrect ones. This stage was crucial for correcting coding errors that could skew the calculations of Classical Test Theory (CTT) indices such as the difficulty index (p), the discrimination index (rpbis), and the KR-20 reliability. Third, response patterns were examined systematically to identify aberrant responding. This meant finding strange or inconsistent answer styles, like random guessing, or a uniform response pattern, for instance, always picking the same option throughout. Also, excessively fast completion times, which may indicate disengagement, were noted. When these patterns appeared, and it was judged that they could threaten the validity of the data, the associated scripts were flagged and removed from the rest of the analysis. Finally, the missing responses within an otherwise proper script were treated in accordance with the usual CTT assumptions. More precisely, all the omitted items were coded as incorrect responses (0). This approach aligns with common large-scale assessment practices, where non-response is treated as a lack of demonstrated knowledge or ability. It also helps keep things consistent when item statistics are computed and prevents an upward tilt in item difficulty estimates. Collectively, these steps of data cleaning and screening strengthened the dataset, ensuring that the psychometric evaluations relied on more accurate, trustworthy, and easily interpretable data.

## Results

**Research Question 1:** What is the trend observed in the difficulty indices of NECO SSCE Mathematics multiple-choice items from 2020 to 2024 among Osun State candidates?

The proportion of candidates who answered each item correctly was used to calculate the item’s difficulty index (p-value) for each examination year. The mean value obtained across all items for each year was computed. The results are presented in Table 1.

**Table 1.** NECO mathematics average difficulty Indices trend (2020–2024).

Year	Mean item difficulty (p̄)	Interpretation
2020	0.75	Very Easy
2021	0.70	Moderately Easy
2022	0.74	Moderately Easy
2023	0.80	Very Easy
2024	0.65	Moderately Easy

Table 1 presents the average item difficulty indices for the NECO Senior School Certificate Examination (SSCE) Mathematics test from 2020 to 2024. The mean item difficulty index (p¯) indicates the percentage of test takers who answered items correctly; higher values indicate easier items, whereas lower values indicate harder items. According to the results, the 2020 NECO Mathematics exam exhibited the greatest mean difficulty index (p¯ = 0.83), implying that the entire set of questions was accessible and straightforward for Osun State students. The data demonstrate that most candidates from that year were successful in answering most test questions. The mean difficulty index for 2021 was 0.70, indicating a test of moderate difficulty, even though the assessment items maintained their appropriate range for large-scale testing. In 2022, item difficulty increased slightly to 0.74, indicating that assessment items from that year were easier to solve than those from 2021. The 2023 examination recorded a further increase in difficulty index to 0.80, indicating that the mathematics items were again largely easy for candidates. The 2024 examination showed a substantial decrease, with a mean difficulty index of 0.65, indicating that this assessment required greater effort from students than in previous years. However, it remained at moderate difficulty levels. The five-year period shows a nonlinear progression, with alternating patterns of test difficulty across examination years. The test forms maintained a consistent level of difficulty, yet their average-difficulty assessments showed irregularities, resulting in testing problems that needed to be resolved across different assessment periods. Score fluctuations between different years require systematic item pretesting and difficulty-balancing methods to establish consistent standards for the NECO Mathematics exams.

**Research Question 2:** How do the NECO SSCE Mathematics items’ discrimination indices vary across the five examination years (2020–2024)?

Table 2 displays the progression of the average item discrimination indices for the NECO SSCE Mathematics exam from 2020 to 2024. The mean discrimination index, calculated using the point-biserial correlation coefficient (rpbis), reflects the extent to which test items distinguish between top-performing and lower-performing students. Higher discrimination values indicate that test items are of higher quality, which helps to correctly rank candidates. The 2020 and 2021 exams produced identical mean discrimination indices of 0.27, indicating moderate tracking ability. The test items from these two years successfully differentiated between candidates with higher and lower abilities, although their effectiveness fell short of the 0.30 benchmark, which defines highly effective test items. The 2022 mean discrimination index rose to 0.29, which indicated a small improvement in item discrimination that approached the standard for high-quality multiple-choice items. The mean discrimination index decreased to 0.25 in 2023, indicating reduced discriminatory power compared with earlier years. The 2023 examination showed reduced effectiveness because a greater number of test items failed to distinguish between high-and low-performing students. The 2024 examination showed a substantial increase in the mean discrimination index, which reached 0.36 and indicated good to very good discrimination power. The 2024 test items were more effective than in previous years at differentiating candidates by ability level. The item discrimination indices exhibit trend patterns over their five-year span, oscillating rather than showing regular development; the data indicate improvements in 2024 after moderate discrimination in previous years. The 2024 data show a significant rise, indicating that either item construction standards improved or items were better evaluated for candidates’ actual skill levels. The annual fluctuations observed by researchers indicate that NECO Mathematics examinations require regular item evaluation and quality assurance procedures to maintain consistent examination performance across years.

**Table 2.** NECO mathematics average discrimination indices trend (2020–2024).

Year	Mean discrimination (rpbis)	Interpretation
2020	0.27	Moderate Index
2021	0.27	Moderate index
2022	0.29	Good
2023	0.25	Weak index
2024	0.36	Very Good Index

**Research Question 3:** What is the extent to which the reliability coefficients of the NECO SSCE Mathematics examinations remain consistent across the years 2020 to 2024?

Table 3 presents the KR-20 statistics for the test batteries of the NECO Senior School Certificate Examination (SSCE) Mathematics over five successive years of administration, from 2020 to 2024. Results for the official outcome indicate that reliability was maintained for the NECO Mathematics exams throughout the five years. More succinctly, KR-20 coefficients were similarly high across all five years: 0.90 in 2020, 0.88 in 2021, 0.87 in 2022, 0.89 in 2023, and 0.90 in 2024. In all cases, values exceeded the minimum acceptable reliability of 0.70, and a majority exceeded 0.90, suggesting high internal consistency. Any mild undulations observed over the year were largely unaddressed, remaining below the required limit, indicating generally consistent, homogeneous functions across items. With KR-20 recovery to.90 in 2024, lying high in its range similar to 2024, our inference regarding the consistency of test construction and the administration of quality assurance processes was justified for NECO by fortuitous excellence.

Hypothesis 1: The difference in item difficulty of NECO SSCE Mathematics examinations between 2020 and 2024 varies significantly.

**Table 3.** NECO mathematics reliability coefficients (kr-20) trend (2020–2024).

Year	KR-20 Reliability
2020	0.90
2021	0.88
2022	0.87
2023	0.89
2024	0.90

The item difficulty indices for NECO SSCE Mathematics items from 2020 to 2024 are presented in Table 4.

**Table 4.** Descriptive statistics of item difficulty of NECO SSCE mathematics between 2020–2024.

Year	N	$\bar{x}$	SD	Min	Max
2020	60	.75	.22773	.00	1.00
2021	60	.70	.23711	.00	.91
2022	60	.74	.14051	.09	.88
2023	60	.80	.16507	.04	.94
2024	60	.65	.19615	.17	.90
Total	300	.73	.20221	.00	1.00

The study uses 60 multiple-choice items each academic year, yielding 300 items across the five testing years. The item difficulty indices range from 0.00 to 1.00 over the five years, with higher values indicating easier assessment materials. The average item difficulty across five years was 0.73 (SD = 0.20), indicating that the NECO SSCE Mathematics assessment materials were of moderate difficulty for students. The data indicate that a considerable number of students answered most test questions correctly during the period examined. The difference in item difficulty over the five years was then assessed using a One-Way Analysis of variance.

In this study, the use of One-Way Analysis of Variance ANOVA was based on the question of whether statistically meaningful differences show up in item-level psychometric indices (for example, item difficulty and item discrimination) across the examination years from 2020 to 2024. In the Classical Test Theory CTT approach, each item is treated as the unit of analysis, and its psychometric characteristics, such as difficulty p and discrimination rpbis, are handled like continuous variables. Because of this, checking the average values of those indices across independent groups, meaning the different years, matches the basic aim of ANOVA, namely testing for mean differences among more than two groups. ANOVA is particularly appropriate here because the research includes five separate groups corresponding to five different years of examination. If we were to perform numerous pairwise t-tests instead, the likelihood of committing a Type I error would increase significantly. In other words, ANOVA provides a more robust and controlled way to detect overall differences among the groups first, before moving on to later post hoc comparisons. The independence of observations assumption was effectively fulfilled since each annual exam used different test forms with separate candidate cohorts, and item statistics were determined independently for each year. Thus, the item-level indices for a particular year are generally independent of those from other years. Concerning the normality assumption, the item-level psychometric measures, mainly difficulty and discrimination scores, are frequently approximately normally distributed when combined over a large number of items, for example, 60 annually in this study. Then, the homogeneity of variance assumption was also looked at. The result is presented in Table 5.

**Table 5.** One-Way ANOVA showing the difference in item difficulty of NECO SSCE mathematics between 2020–2024.

	Sum of Squares	Df	Mean Square	F	Sig.
Between Groups	.806	4	.202	5.206	.000
Within Groups	11.419	295	.039		
Total	12.226	299			

Table 5 presents the results of a one-way Analysis of Variance (ANOVA), which tested whether the NECO SSCE Mathematics item difficulty means differed significantly across examination years from 2020 to 2024. The computed F ratio (F _(4, 7,749)_ = 5.206) was statistically significant at the. 05 level implies that the average item difficulty indices across the five years of examination were statistically significant. In other words, the difficulty levels of NECO SSCE Mathematics test items were not constant in the set range of years of 2020 to 2024, or at least one year’s average item difficulty was statistically significantly different from that of the others. Thus, a Scheffe test was conducted to determine where the difference lies. The results are presented in Table 6.

**Table 6.** Scheffe Multiple comparison of item difficulty of NECO SSCE mathematics between 2020–2024.

(I) Neco Item Difficulty Indices	(J) Neco Item Difficulty Indices	Mean Difference (I-J)	Std. Error	Sig.
2020	2021	.05799	.03592	.626
	2022	.01342	.03592	.998
	2023	−.05094	.03592	.734
	2024	.10131	.03592	.096
2021	2020	−.05799	.03592	.626
	2022	−.04457	.03592	.819
	2023	−.10893	.03592	.059
	2024	.04332	.03592	.834
2022	2020	−.01342	.03592	.998
	2021	.04457	.03592	.819
	2023	−.06436	.03592	.524
	2024	.08789	.03592	.203
2023	2020	.05094	.03592	.734
	2021	.10893	.03592	.059
	2022	.06436	.03592	.524
	2024	.15225 ^*^	.03592	.002
2024	2020	−.10131	.03592	.096
	2021	−.04332	.03592	.834
	2022	−.08789	.03592	.203
	2023	−.15225 ^*^	.03592	.002

Table 6 presents the Scheffe post hoc analysis of item difficulty indices for the mathematics examination in the NECO SSCE for the years 2020–2024. The results indicate that, in the vast majority of pairwise comparisons across the exam years, p-values did not reach the 0.05 significance level. This indicates a general category of item-difficulty consistency over those years. However, a statistically significant difference was observed between the 2023 and 2024 examinations: the mean difference in item difficulty between the two years was estimated at 0.15225 (p = 0.002). This indicates a significant difference in item difficulty between the two years. A positive mean difference indicates that the items of 2023 were relatively easier than the 2024 items (or, otherwise, the 2024 items were harder than the 2023 items).

Hypothesis 2: The item discrimination of NECO SSCE Mathematics examinations between 2020 and 2024 did not vary significantly.

Table 7 presents descriptive statistics of items’ discrimination indices for the NECO SSCE Mathematics for each of the five years from 2020 to 2024. Each year’s examination comprised 60 multiple-choice items, for a total of 300 items analyzed over the five years. The average item discrimination indices ranged from 0.2546 (2023) to 0.3559 (2024). Specifically, 2020 and 2021 had average item discrimination values of 0.2723 and 0.2744, respectively, indicating moderate discrimination, whereas 2022 had a mean item discrimination of 0.2861, indicating a slight improvement in discrimination quality. In 2023, with an average discrimination of 0.2546, items exhibited the lowest differentiation, indicating weaker differentiation power that year. The highest mean discrimination value across all years was 2024, with an average of 0.3559, indicating a substantial improvement in item quality and in items’ ability to differentiate among candidates of varying ability levels.

**Table 7.** Descriptive statistics of item discrimination of NECO SSCE mathematics between 2020–2024.

Year	N	$\bar{x}$	SD	Min	Max
2020	60	.2723	.10728	−.05	.40
2021	60	.2744	.10239	−.05	.42
2022	60	.2861	.07985	.01	.42
2023	60	.2546	.06861	−.06	.36
2024	60	.3559	.22995	−.05	.74
Total	300	.2887	.13489	−.06	.74

According to Table 8, the one-way ANOVA results indicate the possible presence of significant differences in the average discrimination indices for NECO SSCE Mathematics examinations taken between 2020 and 2024. The F-test yielded a significant F-statistic (F = 5.375; p < 0.05). A significant ANOVA result indicates that item discrimination quality differs across at least one year.

**Table 8.** One-Way ANOVA showing the difference in Item discrimination indices of NECO SSCE mathematics between 2020–2024.

	Sum of squares	df	Mean square	F	Sig.
Between Groups	.370	4	.092	5.375	.000
Within Groups	5.071	295	.017		
Total	5.441	299			

Following the detection of a statistically significant source, a Scheffe pairwise comparison was conducted to identify the specific years with notable differences. The results are presented in Table 9.

**Table 9.** Scheffe multiple comparison of item difficulty of NECO SSCE mathematics between 2020–2024.

(I) Neco item difficulty indic	(J) Neco Item difficulty indices	Mean difference (I-J)	Std. Error	Sig.
2020	2021	−.00202	.02394	1.000
	2022	−.01380	.02394	.988
	2023	.01772	.02394	.968
	2024	−.08358 ^*^	.02394	.017
2021	2020	.00202	.02394	1.000
	2022	−.01178	.02394	.993
	2023	.01975	.02394	.954
	2024	−.08156 ^*^	.02394	.022
2022	2020	.01380	.02394	.988
	2021	.01178	.02394	.993
	2023	.03152	.02394	.784
	2024	−.06978	.02394	.078
2023	2020	−.01772	.02394	.968
	2021	−.01975	.02394	.954
	2022	−.03152	.02394	.784
	2024	−.10130 ^*^	.02394	.002
2024	2020	.08358 ^*^	.02394	.017
	2021	.08156 ^*^	.02394	.022
	2022	.06978	.02394	.078
	2023	.10130 ^*^	.02394	.002

Table 9 shows that the year-to-year pairwise analyses yielded few significant results, as indicated by p-values >0.05. Specifically, comparisons between 2020 and 2021, 2020 and 2022, 2022 and 2023, 2021 and 2022, 2021 and 2023, and 2022 and 2023 revealed no significant differences, indicating that test difficulty was stable over time. However, significant differences were observed on the other hand within the 2024 examination year and most of the previous years: significant differences were observed between 2020 and 2024 (Mean Difference = −0.08358, p = .017), 2021 and 2024 (Mean Difference = −0.08156, p = .022), and 2023 and 2024 (Mean Difference = −0.10130, p = .002). This indicated that items in the 2024 examination were significantly more difficult than those in 2020, 2021, and 2023, as indicated by negative mean differences when each year was contrasted with 2024.

## Discussion

The research evaluated changes in psychometric properties of the NECO Senior School Certificate Examination SSCE Mathematics during the five-year period from 2020 to 2024, which tested students from Osun State. The study evaluated changes in psychometric properties of the NECO Senior School Certificate Examination SSCE Mathematics during the five-year period from 2020 to 2024, which tested students from Osun State.

The item difficulty analysis showed that NECO SSCE Mathematics questions had an average difficulty which remained within acceptable CTT limits (p ≈ 0.30–0.80). The descriptive results showed that item difficulty remained stable between 2020 and 2023 until it experienced a significant change in 2024, which ANOVA and Scheffé post-hoc analysis confirmed. The NECO examination established a standard difficulty level which it maintained throughout most years, but the 2024 test showed a clear break from this established pattern. Variations in item difficulty across years of testing are common in large-scale assessments because they reflect changes in educational programs, test developers’ understanding of learning objectives, and the determination of assessment standards, according to. ^19^ The significant difference involving the 2024 items implies a possible recalibration of examination standards, which, while not inherently problematic, underscores the importance of systematic equating and longitudinal monitoring to ensure comparability of scores across years. ^20, 21^

The study found notable variations in item discrimination outcomes when assessed over five separate testing intervals. The NECO Mathematics items achieved good performance in student ability testing because their mean discrimination indices reached acceptable limits which extended to value 0.20. The results showed year-to-year variation in testing results which reached their peak in 2024 when the highest average discrimination value was achieved. The evidence from recent years shows that testing organizations now give better priority to item testing standards which leads to improved assessment results through better testing writing and testing assessment and testing review methods. Assessments for high-stakes testing require high discrimination indices because those indices improve test score interpretation. ^9^ The assessment results show negative discrimination values across multiple years because approximately 10 percent of assessment items did not perform properly because of test item confusion and mistaken answer keys and test item content that did not match test objectives. Developing assessment systems need to conduct regular post-examination item evaluation because previous studies have shown similar results in their research on public examination systems. ^14, 22^

The KR-20 reliability coefficients obtained for the five examination years ranged from 0.87 to 0.90. This range of reliability coefficients confirmed that all test administrations achieved high internal consistency. Manual comparison of the coefficients revealed only minimal year-to-year differences which remained below 0.02. The overall coefficient range between two years was 0.03. The NECO SSCE Mathematics examination maintained consistent measurement accuracy throughout its testing period because these variations stayed within psychometrically nonessential limits. The NECO SSCE Mathematics examination maintained consistent measurement accuracy throughout its testing period because these variations stayed within psychometrically nonessential limits. The test construction practices and test length requirements together with item assessment of the core construct for the test demonstrate reliable assessment through their high reliability coefficients. The study showed stable results which matched the expected standards for large-scale assessments because assessment reliability should remain stable across different testing conditions.

Findings from the study reveal clear changes in how challenging the items seem and in their discrimination indices across the five consecutive years of testing. The 2024 NECO Mathematics exam exhibits notable discrepancies. Rather than merely as statistical quirks, these variations could be seen as signs of broader systemic, curricular, and situational factors influencing both the quality of assessments and candidates’ performance. One possible reason for the swings in how hard each item feels could be how the tests are built, plus how well the content aligns with the blueprint. Studies suggest that if test designers do not follow the blueprint closely and do not balance cognitive demand, the difficulty pattern of items can change substantiall. ^12, 23^ The higher share of hard items in 2024 might mean there was more emphasis on higher-order thinking, or that the item difficulty got calibrated too weakly during development. This matches earlier findings, in which items that were not properly moderated or not given enough tryouts before use can unintentionally become harder than expected. ^24^ When it comes to item discrimination, the observed instability, especially the drop in the more highly discriminating items in 2024, might be signalling trouble with item quality, distractor functioning, or the construct’s representation. Discrimination that is “effective” relies on whether an item can tease apart high from low ability candidates; so, when it declines, it can mean unclear item wording, distractors that do not quite work, or the possibility of multidimensionality. ^25, 26^ This suggests that some of the items used in 2024 may not have measured the latent trait as intended. Beyond considerations of test construction, changes in curriculum and exam reforms provide a valuable perspective on the situation. This is in line with a study arguing that changes in educational policies, such as curriculum modifications or adjustments in instructional priorities, can create a mismatch between classroom content and assessment content, thereby affecting the difficulty and effectiveness of test items. ^27^ If the 2024 examination mirrored updated curricular expectations but there were no corresponding changes in day-to-day teaching practices, the candidates might have ended up not adequately prepared for the content that appeared.

A pretty compelling account emerges in the post-pandemic educational landscape. Disruptions in teaching and learning due to the COVID-19 pandemic led to reduced instructional hours, gaps in curriculum coverage, and learning setbacks. ^28, 29^ A study observed significant declines in students’ academic preparedness and increased variation in their performance following the pandemic. ^20^ So, for the 2024 cohort, because they had longer interruptions during key learning stretches, they may end up with a thinner grasp of basic mathematical ideas, which would help explain why items feel more difficult and why discrimination indices are lower. Also, these findings might be tied to problems with test-form comparability from one year to the next, as psychometric papers repeatedly note. When there are no formal equating procedures, any shifts you see in item parameter estimates between test administrations may not only mean the items have changed in quality, but could also come from differences in what each cohort can do. In other words, the observed patterns may reflect variation in cohort ability more than anything else. ^20, 30^ So, it becomes really important to demonstrate measurement equivalence before making comparisons across years. Generally, the inconsistencies detected mainly in 2024 seem to stem from a combination of test design variables, extensive changes in education policies, and cohort-specific factors. This underscores the need for enhanced test development procedures, such as pretesting items, calibration, and equating, coupled with ongoing synchronisation among curriculum, instructional methods, and assessment efforts.

### Implications for examination quality assurance

The research results demonstrate that NECO SSCE Mathematics exam has maintained its strong reliability throughout testing while its testing materials show acceptable quality standards. The test development process demonstrates its dynamic nature through ongoing need for psychometric assessments which experts should conduct to maintain valid results in high-stakes certification and selection exams. ^19, 21^ Recommend that regular item analysis, alongside structured feedback loops for item writers and moderators, would help sustain improvements in discrimination quality while ensuring that changes in difficulty do not compromise fairness or comparability across cohorts. Such practices are critical for strengthening public confidence in examination outcomes and supporting evidence-based assessment reforms in Nigeria.

## Conclusion

The study investigated the psychometric evolution of the NECO Senior School Certificate Examination (SSCE) Mathematics test over a five-year period starting from 2020 to 2024 for candidates in Osun State through the application of Classical Test Theory. The research results demonstrate that examination items in high-stakes public assessments maintain consistent quality while exhibiting different levels of performance. The study concludes that NECO SSCE Mathematics test demonstrates strong psychometric properties which show particular excellence in testing reliability. The examination requires continuous systematized monitoring of item difficulty and discrimination assessment which will enable fair testing and consistent evaluation of academic performance across different years.

### Recommendations

Based on the findings of this study, the following recommendations are made:

i. **Routine Post-Examination Item Analysis:** NECO should institutionalize comprehensive post-examination item analysis after each examination cycle to identify poorly functioning items, particularly those with low or negative discrimination indices, for revision or elimination.
ii. **Strengthening Item Writer Training:** Regular capacity-building workshops should be organized for item writers and moderators, with emphasis on writing items that achieve optimal difficulty and high discrimination in line with Classical Test Theory guidelines.
iii. **Monitoring Longitudinal Item Trends:** NECO should adopt a structured framework for monitoring longitudinal trends in psychometric indices to ensure consistency of examination standards across years and prevent unintended shifts in difficulty.
iv. **Use of Statistical Evidence in Test Review:** Decisions regarding item retention, modification, or replacement should be guided by empirical psychometric evidence rather than solely by expert judgment.
v. **Expansion to Advanced Psychometric Models:** Future evaluations of NECO examinations should complement Classical Test Theory with Item Response Theory analyses to provide deeper insights into item functioning and candidate ability estimation.
vi. **Policy Support for Examination Quality Assurance:** Educational policymakers should support the integration of psychometric research findings into national examination quality assurance policies to enhance public confidence in examination results.

## Ethical approval

Ethical approval was not required for this study as it involved secondary analysis of anonymized examination data with no direct involvement of human participants.

## Data Availability

Open Science Framework: Adediwura, A. A., Babayemi, B. O., & Odumbo, O. I. (2026, May 12). Trends in the Psychometric Characteristics of NECO Mathematics Senior School Certificate Examination Over a Period of Five Years (2020–2024) among Osun State’s Candidates. https://doi.org/10.17605/OSF.IO/GSPU5 ^31^ This project contains the following extended data
• ODUMBO DATA REPOSITORY file.pdf/doc. Data are available under the terms of the Creative Commons Zero “No rights reserved” data waiver (CC0 1.0 Public domain dedication).

## References

[1] Aborisade OJ Fajobi OO : Comparative analysis of psychometric properties of mathematics items constructed by WAEC and NECO in Nigeria using item response theory approach. *Educ Res Rev.* 2020;15(1):1–7. 10.5897/ERR2019.3850

[2] Oghenerume RA : Item statistics disparity between 2023 WASSCE and NECO SSCE mathematics large-scale assessments. *Int J Educ Res.* 2025;16(1).

[3] Jimoh K Opesemowo AA Faremi YA : Psychometric analysis of SSCE 2017 NECO English language multiple choice items using IRT. *J Appl Res Multidiscip Stud.* 2022.

[4] Adediwura AA Asowo PA : Examining the nature of item bias on NECO mathematics senior school certificate dichotomously scored items in Nigeria. *Int J Contemp Educ.* 2022.

[5] OECD: An OECD learning framework 2030. *The future of education and labor.* Cham: Springer International Publishing;2019; pp.23–35. 10.1007/978-3-030-26068-2_3

[6] Kane MT : Validating the interpretations and uses of test scores. *J Educ Meas.* 2021;58(2):135–150.

[7] De Ayala RJ : *The theory and practice of item response theory.* 2nd ed. New York: Guilford Press;2013.

[8] Crocker L Algina J : *Introduction to classical and modern test theory. * 2nd ed. Boston: Cengage Learning;2018.

[9] Downing SM : Reliability: On the reproducibility of assessment data. *Med Educ.* 2003;37(9):830–837. 15327684 10.1111/j.1365-2929.2004.01932.x

[10] Bond TG Fox CM : *Applying the Rasch model: Fundamental measurement in the human sciences.* 3rd ed. New York: Routledge;2015.

[11] Kline RB : *Principles and practice of structural equation modeling.* 4th ed. New York: Guilford Press;2016.

[12] Lane S Raymond MR Haladyna TM : *Handbook of test development.* 2nd ed. New York: Routledge;2016.

[13] Aborisade OJ Fajobi OO : Comparative analysis of psychometric properties of mathematics items constructed by WAEC and NECO in Nigeria using item response theory approach. *Educ Res Rev.* 2020;15(1):1–7. 10.5897/ERR2019.3850

[14] Jimoh MA Yusuf A Adebayo SO : Dimensionality and item functioning of public examination items in Nigeria. *J Educ Meas Eval.* 2022;14(2):45–62.

[15] Ekong EM Ubi IO Eni EI : Differential item functioning of 2018 basic education certificate examination (BECE) in mathematics: A comparative study of male and female candidates.

[16] Adeyemi AA Arogundade I Oluwakemi BBO : Hybrid learning approaches and their effect on students’ engagement and academic performance in secondary schools in some Nigerian states. *Int J Res Innov Soc Sci.* 2025;9(11).

[17] Zumbo BD : A measure of fairness: Using differential item functioning to detect bias. *Educ Meas Issues Pract.* 2016;35(1):3–12.

[18] Millsap RE : *Statistical approaches to measurement invariance.* New York: Routledge;2018.

[19] Haladyna TM Rodriguez MC Downing SM : A review of multiple-choice item-writing guidelines for classroom assessment. *Appl Meas Educ.* 2018;31(1):1–18.

[20] Kolen MJ Brennan RL : *Test equating, scaling, and linking.* 3rd ed. New York: Springer;2014. 10.1007/978-1-4939-0317-7

[21] Kim S Lee WC : Trends in item difficulty and discrimination across repeated large-scale assessments. *Educ Meas Issues Pract.* 2021;40(3):23–34.

[22] Awopeju OA Afolabi ERI : Comparative analysis of classical test theory and item response theory-based item parameter estimates of senior school certificate mathematics examination. *Eur Sci J.* 2016;12(28):263–284.

[23] Haladyna TM Rodriguez MC : *Developing and validating test items.* 4th ed. Routledge;2019.

[24] Adedoyin OO Mokobi T : Using IRT psychometric analysis in examining the quality of multiple-choice examination items. *Int J STEM Educ.* 2019;6(1):1–14.

[25] Rodriguez MC : Three options are optimal for multiple-choice items: A meta- analysis of 80 years of research. *Educ Meas: Issues Pract.* 2016;35(2):37–50.

[26] Tarrant M Ware J Mohammed AM : An assessment of functioning distractors in multiple-choice questions: A descriptive analysis. *BMC Med Educ.* 2020;20(1):1–8. 10.1186/1472-6920-9-40 PMC2713226 19580681

[27] UNESCO: *Education in a post-COVID world: Nine ideas for public action.* UNESCO Publishing;2021.

[28] Dorn E Hancock B Sarakatsannis J : *COVID-19 and education: The lingering effects of unfinished learning.* McKinsey & Company Report;2021.

[29] Engzell P Frey A Verhagen M : Learning loss due to school closures during the COVID-19 pandemic. *Proc Natl Acad Sci USA.* 2021;118(17):e2022376118. 10.1073/pnas.2022376118 33827987 PMC8092566

[30] Von Davier AA : *Statistical models for test equating, scaling, and linking.* Springer;2018.

[31] Adediwura AA Babayemi BO Odumbo OI : Trends in the Psychometric Characteristics of NECO Mathematics Senior School Certificate Examination Over a Period of Five Years (2020–2024) among Osun State’s Candidates. 2026, May 12. 10.17605/OSF.IO/GSPU5

