# Supplementary material for: Trends in the Psychometric Characteristics of NECO Mathematics Senior School Certificate Examination Over a Period of Five Years (2020-2024) among Osun State Candidates, Nigeria
Source: F1000Res. 2026 Jul 11;15:818. Originally published 2026 May 27. [Version 2] doi: 10.12688/f1000research.182391.2 (PMC13555035; doi:10.12688/f1000research.182391.2)
Supplement: Peer review history

# Reviewer report: Reviewer response for version 2

**DOI:** 10.5256/f1000research.205217.r500522

**Published:** 2026-07-24

**Authors:** Omale Onuh

**Affiliations:** 1 Joseph Sarwuan Tarka University, Makurdi, Nigeria

Article is ok now for indexing.

Is the work clearly and accurately presented and does it cite the current literature?

Yes

If applicable, is the statistical analysis and its interpretation appropriate?

No

Are all the source data underlying the results available to ensure full reproducibility?

No

Is the study design appropriate and is the work technically sound?

No

Are the conclusions drawn adequately supported by the results?

Yes

Are sufficient details of methods and analysis provided to allow replication by others?

No

Reviewer Expertise:

Educational Measurment and Evaluation, Bias in Psychometric

I confirm that I have read this submission and believe that I have an appropriate level of expertise to confirm that it is of an acceptable scientific standard.

# Reviewer report: Reviewer response for version 2

**DOI:** 10.5256/f1000research.205217.r501098

**Published:** 2026-07-22

**Authors:** Torang Siregar

**Affiliations:** 1 Department of Mathematics Education, PhD Student in Mathematics Education, State University of Padang, Padang, West Sumatra, Indonesia

**1.**

**Strengths:**

1. The revised version (V2) demonstrates substantial improvement, with expanded Introduction and Discussion sections incorporating recent literature
2. Authors have responded to reviewer concerns about equating, with a clear acknowledgment of the absence of formal test equating
3. References include relevant psychometric works and recent Nigerian examination studies

**Critical Issues:**

**a) Table Reference and Data Inconsistencies:**

1. Table 1 shows mean item difficulty values (0.75, 0.70, 0.74, 0.80, 0.65) but the interpretation column labels 2020 as "Very Easy" while the actual value (0.75) suggests a moderate-to-easy range
2. The decimal place formatting is inconsistent: Table 1 shows "0.75" while Table 4 shows "0.75" with additional decimal places that appear corrupted (e.g., ".22773")
3. Table 4 contains corrupted decimal formatting: ".22773" and ".14051" appear incorrectly formatted; "1.09" shows a misplaced decimal
4. The total N in Table 4 is listed as 300, but according to the text, each year has 60 items, and there are five years: 5 × 60 = 300. This is correct.

**b) Interpretation Inconsistencies:**

1. Table 1 describes 2020 as "Very Easy" (p=0.75), but the text states "the 2020 NECO Mathematics exam exhibited the greatest mean difficulty index (p̄ = 0.83)"—this is a discrepancy (0.75 vs 0.83)
2. The abstract states "item difficulty remained largely stable over the years, except in the most recent examination year," which aligns with the ANOVA findings (only 2023 vs 2024 was significant)

**c) Literature Coverage:**

1. The discussion of post-pandemic effects (references 26-29) adds valuable context
2. However, some claims still lack citation: "Studies suggest that if test designers do not follow the blueprint closely..." (page 12) should have a specific citation

**Required Actions:**

1. Correct all decimal formatting in Table 4
2. Reconcile the discrepancy between Table 1 (0.75) and text (0.83) for the 2020 difficulty index
3. Add specific citations for claims about test blueprint adherence and item difficulty variation
4. Ensure all references are properly cited and formatted

***2.***

***Critical Issues:***

***a) Missing Test Equating:***

*This is the most significant methodological concern. The authors compare psychometric indices across five different examination years but:*

1. *No anchor items, linking designs, or equating procedures are reported*
2. *Different test forms were administered each year to different candidate cohorts*
3. *Without equating, observed differences may reflect form difficulty rather than changes in examination quality*

***What the Authors Did:***

*The authors have acknowledged this limitation in the revised version (V2) and added a section stating: "Although this study did not employ formal test equating, its longitudinal analyses remain appropriate for evaluating trends in examination quality" and "the findings should be interpreted as indicators of potential changes in test quality rather than definitive differences in examination difficulty or candidate performance."*

***Assessment:***

*While the authors have appropriately acknowledged the limitation, this does not fully resolve the technical concern. For a study titled "Trends," readers reasonably expect some method of placing scores or item parameters on a common scale. The lack of equating compromises the validity of the trend conclusions.*

***b) Unit of Analysis Mismatch with ANOVA:***

1. *The study uses item-level psychometric indices (difficulty and discrimination) as the unit of analysis*
2. *ANOVA is applied to these indices (n=60 per year, total n=300)*
3. *However, the authors state: "the independence of observations assumption was effectively fulfilled since each annual exam used different test forms with separate candidate cohorts"*
4. *This is questionable: even with different cohorts, item statistics from the same year are not truly independent because they share common candidates and test context*
5. *Additionally, the F-statistic in Table 5 shows F(4,749) = 5.206, which suggests df error = 749, but with 300 items total and 5 groups, df error should be 295 (300-5). The reported df (4,749) suggests a different unit of analysis was used*

***c) Classification Criteria Not Fully Justified:***

1. *The authors categorize discrimination indices as "Moderate" (0.27), "Good" (0.29), "Weak index" (0.25), "Very Good Index" (0.36) in Table 2*
2. *However, the criteria for these classifications are not clearly specified*
3. *Standard thresholds (e.g., Ebel's criteria: >0.40 excellent, 0.30-0.39 good, 0.20-0.29 fair, <0.20 poor) are not referenced*

***Required Actions:***

1. *Provide a more detailed justification for comparing indices without equating, or consider reframing the analysis as a descriptive quality monitoring study rather than a "trend" study*
2. *Clarify the exact unit of analysis in the ANOVA and ensure the degrees of freedom are correct*
3. *Specify the classification criteria for difficulty and discrimination indices with citations (e.g., Ebel, 1972; Crocker & Algina, 2008)*
4. *Consider providing item-level distributions rather than only mean values to better illustrate the data*

**3.**

**Strengths:**

1. The authors have expanded the Methodology section in V2
2. Data cleaning procedures are now described (10% missing responses excluded)
3. Missing responses coded as incorrect is explicitly stated
4. The response to reviewers indicates that statistical software has been specified

**Critical Issues:**

**a) Missing Specific Information:**

1. The statistical software used is not clearly identified in the manuscript itself (the authors mention it was added but it's not visible in the provided PDF)
2. No mention of software versions
3. No formula for the point-biserial correlation coefficient (rpbis) is provided
4. The procedure for aggregating item-level data to compute overall test reliability (KR-20) is not described

**b) Missing Data Handling Ambiguity:**

1. The authors state: "the missing responses within an otherwise proper script were treated in accordance with the usual CTT assumptions. More precisely, all omitted items were coded as incorrect responses (0)"
2. However, it's unclear how this was handled for the calculation of item difficulty: were omitted responses included in the denominator when computing p-values?
3. This is important because coding omissions as incorrect could artificially deflate difficulty estimates

**c) Sampling Details:**

1. The authors used proportional random sampling (10% of total population)
2. It's unclear how the random selection was actually conducted from NECO records
3. No description of the randomization procedure

**Required Actions:**

1. Clearly specify the statistical software, version, and all analysis parameters
2. Provide the formulas used for item difficulty (p), item discrimination (rpbis), and KR-20 reliability
3. Describe exactly how missing responses were handled in the computation of each index
4. Specify the randomization method for sampling
5. Provide the classification criteria for item difficulty and discrimination with citations

***4.***

***Strengths:***

1. *Use of one-way ANOVA for comparing group means is appropriate in principle*
2. *Scheffe post-hoc test is a conservative choice appropriate for unequal group comparisons*
3. *The authors have provided a justification for using ANOVA in the revised manuscript*

***Critical Issues:***

***a) Degrees of Freedom Mismatch:***

- *Table 5 reports: F(4, 749) = 5.206*
- *With 300 items total (60 per year × 5 years), the degrees of freedom should be:*
  1. *Between groups: k-1 = 4*
  2. *Within groups: N-k = 300-5 = 295*
  3. *Total: N-1 = 299*
- *The reported df error of 749 suggests either:*
  1. *A different unit of analysis was used (possibly item × candidate level)*
  2. *The values in the table are incorrect*
  3. *There is a reporting error*
- *This is a serious concern that the authors must address*

***b) ANOVA Assumptions:***

1. *The authors discuss normality: "item-level psychometric measures... are frequently approximately normally distributed"*
2. *However, no formal tests of normality are reported (e.g., Shapiro-Wilk, Kolmogorov-Smirnov)*
3. *The homogeneity of variance assumption is mentioned but no test results (e.g., Levene's test) are reported*
4. *Difficulty indices (proportions) are bounded between 0 and 1 and may not satisfy the normality assumption, especially if distributions are skewed*

***c) Interpretation of Statistical Significance:***

1. *The authors correctly identify that the 2023-2024 difference in difficulty was significant (p = 0.002, mean difference = 0.152)*
2. *However, the practical significance of this difference should be discussed: is a 0.15 increase in p-value meaningful in an examination context?*
3. *The effect size is not reported (e.g., Cohen's d, eta-squared)*
4. *For discrimination indices, the ANOVA is significant (F = 5.375, p < 0.05), but post-hoc results are not shown*
5. *The authors note negative discrimination values but do not explain how items with negative discrimination should be interpreted or handled*

***d) Reliability Interpretation:***

1. *The KR-20 coefficients (0.87-0.90) are correctly interpreted as high internal consistency*
2. *However, the authors state: "KR-20 recovery to 0.90 in 2024, lying high in its range similar to 2024" — this appears to be an editing error or unclear phrasing*
3. *The slight variations in reliability (range 0.03) are correctly described as negligible*

***Required Actions:***

1. *Correct the ANOVA degrees of freedom or clarify the unit of analysis*
2. *Report assumption checks (normality tests, Levene's test) for the ANOVA*
3. *Report effect sizes for significant ANOVA results*
4. *Report the full Scheffe post-hoc results for discrimination indices (currently only difficulty is shown in Table 6)*
5. *Discuss the practical significance of the observed differences*
6. *Explain how items with negative discrimination are interpreted in practice*

**5.**

**Strengths:**

1. The authors have deposited data in the Open Science Framework (OSF)
2. DOI: https://doi.org/10.17605/OSF.IO/GSPU5
3. Data are available under CC0 1.0 Public Domain Dedication

**Critical Issues:**

- The OSF repository is described as containing "ODUMBO DATA REPOSITORY file.pdf/doc"
- It is unclear whether this contains:
  1. Raw item-level response data
  2. Computed psychometric indices
  3. Statistical analysis outputs
  4. Complete dataset with all variables
- The authors state "the source data underlying the results are available," but the specific contents are not described

**Required Actions:**

1. Clearly describe what is contained in the OSF repository
2. Ensure all files needed to reproduce the analysis are accessible (cleaned data, item-level indices, analysis scripts if applicable)
3. Use a consistent naming convention and provide a README file

***6.***

***Strengths:***

1. *Conclusions about high reliability are well-supported by KR-20 coefficients*
2. *Conclusion about the 2024 difficulty change is supported by the Scheffe post-hoc test*
3. *Recommendations are reasonable and practical*

***Critical Issues:***

***a) Overstatement of "Stable" Difficulty:***

*The authors conclude: "item difficulty remained largely stable over the years, except in the most recent examination year." However:*

1. *Table 1 shows variations: 0.75, 0.70, 0.74, 0.80, 0.65*
2. *The range from 0.65 to 0.80 is substantial (a 0.15 difference, or a 23% change relative to the 2024 value)*
3. *The ANOVA found no significant differences among most years, but this may reflect low statistical power (n=60 per group) rather than genuine stability*
4. *The conclusion should more accurately reflect that difficulty varied, with only one pairwise comparison reaching statistical significance*

***b) Limited Generalizability:***

1. *The study is limited to Osun State candidates, but the title suggests a broader focus*
2. *The authors state: "This limitation of scope is important because Osun State candidates may not accurately represent the performance patterns of students in other parts of the country"*
3. *However, this is acknowledged in the manuscript, which is appropriate*

***c) No Validity Evidence:***

1. *The study focuses on reliability, difficulty, and discrimination*
2. *However, no validity evidence is provided (e.g., content validity, criterion-related validity, construct validity)*
3. *The authors use the term "psychometric characteristics" but do not address validity, which is a core psychometric property*

***d) Unsupported Claim about Improved Item Writing:***

1. *The authors state: "The evidence from recent years shows that testing organizations now give better priority to item testing standards which leads to improved assessment results through better testing writing"*
2. *This is speculative and not directly supported by the data*
3. *The improvement in discrimination in 2024 could be due to other factors*

***Required Actions:***

1. *Reframe the conclusion about "stability" to acknowledge the observed variation and the statistical findings more precisely*
2. *Acknowledge the lack of validity evidence as a limitation*
3. *Avoid causal claims about improvements in item writing without direct evidence*
4. *Strengthen the discussion of generalizability limitations*

***General Comments on the Revision***

*The authors have clearly made significant efforts to address reviewer comments in Version 2 (V2). The response to reviewers shows careful attention to each point raised. Specific improvements include:*

***Addressed:***

1. *Expanded Methodology section with more detail on item analysis procedures*
2. *Clarification of the Classical Test Theory framework*
3. *Acknowledgment of the absence of formal test equating*
4. *Expanded Discussion with consideration of curriculum changes, post-pandemic effects, and examination reforms*
5. *Correction of year coding errors*
6. *Revision of objectives to match analyses (removal of distractor efficiency)*

***Remaining Concerns:***

1. *The ANOVA degrees of freedom issue remains unresolved*
2. *The decimal formatting in Table 4 still appears corrupted*
3. *Software and analysis parameters need to be more clearly specified in the manuscript itself*
4. *Classification criteria for psychometric indices need explicit citation*

**Overall Recommendation: Approved with Reservations**

 The manuscript addresses an important and timely topic in educational measurement and assessment. The large dataset (21,175 candidates) and five-year timeframe provide substantial empirical value. The authors have responded thoughtfully to earlier reviewer concerns and made meaningful improvements.

 However, several technical issues must be addressed to make the article scientifically sound:

**Must Address:**

1. *Correct the ANOVA degrees of freedom – The reported df error (749) with 300 items is impossible. Clarify whether a different unit of analysis was used or correct the reporting.*
2. *Fix formatting errors in Table 4 – The decimal values appear corrupted (e.g., ".22773", ".14051") and need correction.*
3. *Reconcile the discrepancy between Table 1 (0.75) and text (0.83) for the 2020 difficulty index.*
4. *Report post-hoc results for discrimination indices – Table 6 only shows difficulty post-hoc. The ANOVA for discrimination was significant, so post-hoc results should be reported.*
5. *Specify classification criteria with citations – Clarify what thresholds define "Very Easy," "Moderate," "Good," "Weak," and "Very Good" discrimination.*
6. *Provide software and parameter details – Specify exact software, versions, and analysis parameters in the manuscript.*

**Recommended Improvements:**

1. *Report effect sizes for the ANOVA results (eta-squared or partial eta-squared).*
2. *Discuss practical significance alongside statistical significance for the 2023-2024 difficulty difference (mean difference = 0.152).*
3. *Describe OSF repository contents in detail to ensure full reproducibility.*
4. *Acknowledge the lack of validity evidence as a limitation of the study.*
5. *Reframe the "stability" conclusion to accurately reflect the observed variations in difficulty.*

**Additional Minor Comments**

1. ***Language editing:*** *While the V2 revision shows improvement, there are still some awkward constructions and errors (e.g., "the test forms maintained a consistent level of difficulty, yet their average-difficulty assessments showed irregularities, resulting in testing problems that needed to be resolved across different assessment periods"). Further editing would enhance clarity.*
2. ***Reference consistency:*** *Some references appear with incomplete formatting (e.g., reference 16 on page 14 appears incomplete).*
3. ***Table formatting:*** *The use of decimal points and commas is inconsistent (e.g., Table 1 shows "0.75" while Table 4 shows ".75").*
4. ***Duplicate text:*** *The manuscript has duplicate sentences in some places (e.g., "The NECO SSCE Mathematics examination maintained consistent measurement accuracy throughout its testing period because these variations staved within psychometrically nonessential limits" appears twice with slight variations).*

**Final Assessment**

 The authors have made substantial progress in revising this manuscript, and the topic is clearly relevant to educational measurement and assessment practice in Nigeria. With the corrections outlined above, particularly the ANOVA degrees of freedom and formatting errors, the manuscript would be suitable for indexing. The study provides valuable longitudinal psychometric evidence that can inform examination quality assurance practices.

Is the work clearly and accurately presented and does it cite the current literature?

Partly

If applicable, is the statistical analysis and its interpretation appropriate?

Partly

Are all the source data underlying the results available to ensure full reproducibility?

Partly

Is the study design appropriate and is the work technically sound?

Partly

Are the conclusions drawn adequately supported by the results?

Partly

Are sufficient details of methods and analysis provided to allow replication by others?

Partly

Reviewer Expertise:

Mathematics Education; STEM Education; RME; AI in Education, Mathematics Teaching and Learning; Educational Technology; GeoGebra-Assisted Learning; Artificial Intelligence in Education; Mathematical Literacy; Problem Solving; Critical Thinking; Numeracy; Curriculum and Instruction; Teacher Education; Research & Development; Higher Education; Social Science.

I confirm that I have read this submission and believe that I have an appropriate level of expertise to confirm that it is of an acceptable scientific standard, however I have significant reservations, as outlined above.

# Reviewer report: Reviewer response for version 2

**DOI:** 10.5256/f1000research.205217.r500521

**Published:** 2026-07-15

**Authors:** Musa Adekunle Ayanwale

**Affiliations:** 1 University of Pretoria, Pretoria, South Africa

All earlier concerns have been addressed. Thank you.

Is the work clearly and accurately presented and does it cite the current literature?

Partly

If applicable, is the statistical analysis and its interpretation appropriate?

No

Are all the source data underlying the results available to ensure full reproducibility?

Partly

Is the study design appropriate and is the work technically sound?

No

Are the conclusions drawn adequately supported by the results?

Partly

Are sufficient details of methods and analysis provided to allow replication by others?

No

Reviewer Expertise:

Test development, Psychometrics, Test Theories, Educational Measurement.

I confirm that I have read this submission and believe that I have an appropriate level of expertise to confirm that it is of an acceptable scientific standard.

# Reviewer report: Reviewer response for version 1

**DOI:** 10.5256/f1000research.201328.r489954

**Published:** 2026-06-13

**Authors:** Musa Adekunle Ayanwale

**Affiliations:** 1 University of Pretoria, Pretoria, South Africa

The topic is important because monitoring psychometric quality is essential for maintaining fairness, reliability, and public confidence in high-stakes examinations. The study benefits from a large dataset and addresses a relevant assessment issue. However, the manuscript contains several methodological, psychometric, statistical, and reporting weaknesses that substantially limit confidence in the conclusions.

 The most significant concern is comparing psychometric indices across different examination years without evidence that the examination forms are psychometrically comparable. The authors do not report any equating procedures, anchor items, linking design, or measurement invariance analyses. Consequently, differences observed across years may reflect differences in examination forms rather than genuine changes in examination quality. This limitation affects the validity of the reported trend analyses and should be explicitly addressed.

 The methodology section lacks sufficient detail to permit replication. Important information is missing regarding item analysis procedures, the computation of difficulty and discrimination indices, the treatment of missing responses, the statistical software used, classification criteria for psychometric indices, and data cleaning procedures. These omissions reduce transparency and reproducibility.

 The statistical analyses also require reconsideration. The rationale for applying ANOVA to item-level psychometric indices is not adequately justified, assumptions are not discussed, and several reporting inconsistencies are present. For example, some tables refer to 2025 rather than 2024, and discrepancies exist between ANOVA values reported in the text and those reported in the tables. These issues raise concerns regarding the accuracy of the analyses and interpretation.

 Furthermore, the study relies exclusively on Classical Test Theory despite extensive discussion of Item Response Theory and Differential Item Functioning in the literature review. Additional psychometric evidence relating to dimensionality, item fit, local independence, validity, and fairness would strengthen the study considerably. There is also a mismatch between the stated objectives and the reported results: distractor efficiency is listed as a psychometric characteristic of interest, yet no distractor analysis is presented.

 The discussion section primarily repeats the results rather than providing a deeper interpretation. Greater engagement with the literature is needed to explain possible reasons for the observed fluctuations in difficulty and discrimination, particularly the notable changes reported for the 2024 examination. Consideration of curriculum changes, examination reforms, post-pandemic educational effects, and differences in candidate preparation would strengthen the discussion.

Is the work clearly and accurately presented and does it cite the current literature?

Partly

If applicable, is the statistical analysis and its interpretation appropriate?

No

Are all the source data underlying the results available to ensure full reproducibility?

Partly

Is the study design appropriate and is the work technically sound?

No

Are the conclusions drawn adequately supported by the results?

Partly

Are sufficient details of methods and analysis provided to allow replication by others?

No

Reviewer Expertise:

Test development, Psychometrics, Test Theories, Educational Measurement.

I confirm that I have read this submission and believe that I have an appropriate level of expertise to state that I do not consider it to be of an acceptable scientific standard, for reasons outlined above.

# Response

**Published:** 2026-06-29

**Authors:** Odunayo Odumbo

**Affiliations:** Humanities, Kampala International University - Western Campus, Bushenyi, Western Region, Uganda

Reviewer comment - **Musa Adekunle Ayanwale**

**Reviewer comment: **The topic is important because monitoring psychometric quality is essential for maintaining fairness, reliability, and public confidence in high-stakes examinations. The study benefits from a large dataset and addresses a relevant assessment issue. However, the manuscript contains several methodological, psychometric, statistical, and reporting weaknesses that substantially limit confidence in the conclusions.

***Author's Comments:** Thank you for your valuable comments. The Methodology section has been expanded to provide sufficient detail to facilitate the replication of the study. Specifically, additional information has been included regarding the procedures used for item analysis, the computation of psychometric indices, the handling of missing data, and the criteria adopted for classifying item difficulty and discrimination levels. Furthermore, the statistical software used for data analysis has been clearly specified and described in the revised manuscript.*

**Reviewer comment: **The most significant concern is comparing psychometric indices across different examination years without evidence that the examination forms are psychometrically comparable. The authors do not report any equating procedures, anchor items, linking design, or measurement invariance analyses. Consequently, differences observed across years may reflect differences in examination forms rather than genuine changes in examination quality. This limitation affects the validity of the reported trend analyses and should be explicitly addressed.

***Author’s Comment:** Thank you for this insightful and important comment. We agree that comparisons of psychometric indices across different examination years should be interpreted with caution in the absence of formal equating procedures, anchor items, linking designs, or measurement invariance analyses.*

**Reviewer comment: **The methodology section lacks sufficient detail to permit replication. Important information is missing regarding item analysis procedures, the computation of difficulty and discrimination indices, the treatment of missing responses, the statistical software used, classification criteria for psychometric indices, and data cleaning procedures. These omissions reduce transparency and reproducibility.

***Author's Comments:** Thank you for your valuable comments. The Methodology section has been expanded to provide sufficient detail to facilitate the replication of the study. Specifically, additional information has been included on the procedures used for item analysis, the computation of psychometric indices, the handling of missing data, and the criteria adopted for classifying item difficulty and discrimination indices. Furthermore, the statistical software used for data management and analysis has been clearly specified in the revised manuscript.*

**Reviewer comment: **The statistical analyses also require reconsideration. The rationale for applying ANOVA to item-level psychometric indices is not adequately justified, assumptions are not discussed, and several reporting inconsistencies are present. For example, some tables refer to 2025 rather than 2024, and discrepancies exist between ANOVA values reported in the text and those reported in the tables. These issues raise concerns regarding the accuracy of the analyses and interpretation.

***Author's Comments:** Thank you for your valuable observation. The revised manuscript now provides a clear justification for the use of one-way ANOVA, explaining its suitability for comparing psychometric indices across multiple examination years and determining whether statistically significant differences existed among the examination cohorts.*

**Reviewer comment: **Furthermore, the study relies exclusively on Classical Test Theory despite extensive discussion of Item Response Theory and Differential Item Functioning in the literature review. Additional psychometric evidence relating to dimensionality, item fit, local independence, validity, and fairness would strengthen the study considerably. There is also a mismatch between the stated objectives and the reported results: distractor efficiency is listed as a psychometric characteristic of interest, yet no distractor analysis is presented.

***Author's Comments:** Thank you for your valuable observation. Although Item Response Theory (IRT) and Differential Item Functioning (DIF) were reviewed in the literature, the present study was intentionally designed within the Classical Test Theory (CTT) framework. The revised manuscript now explicitly acknowledges this methodological scope and discusses it as a limitation, while recommending the application of IRT- and DIF-based analyses in future studies to provide more robust psychometric evaluation and cross-year comparability.*

**Reviewer comment: **The discussion section primarily repeats the results rather than providing a deeper interpretation. Greater engagement with the literature is needed to explain possible reasons for the observed fluctuations in difficulty and discrimination, particularly the notable changes reported for the 2024 examination. Consideration of curriculum changes, examination reforms, post-pandemic educational effects, and differences in candidate preparation would strengthen the discussion.

***Author's Comments:** Thank you for your valuable observation. The Discussion section has been revised to consider the potential influence of curriculum modifications, examination reforms, changes in candidate preparation, and post-pandemic educational disruptions on the interpretation of the study findings. These additions provide a broader contextual framework for the observed psychometric trends and strengthen the discussion of their implications.*

# Reviewer report: Reviewer response for version 1

**DOI:** 10.5256/f1000research.201328.r489957

**Published:** 2026-06-03

**Authors:** Omale Onuh

**Affiliations:** 1 Joseph Sarwuan Tarka University, Makurdi, Nigeria

Despite these strengths, the manuscript requires substantial revision before it can be considered suitable for indexing. Several methodological, statistical, conceptual, and presentation issues need to be addressed. First, there is a mismatch between the stated objectives and the analyses conducted. While distractor efficiency was included among the psychometric characteristics to be examined, no results or analyses relating to distractor efficiency were presented. The authors should either provide the relevant analyses or revise the objectives accordingly.

 The methodology section lacks sufficient detail to allow replication of the study. Important information regarding the procedures used for item analysis, computation of psychometric indices, handling of missing data, criteria for classifying item difficulty and discrimination levels, and statistical software employed is not adequately described. Furthermore, the study compares psychometric characteristics across different examination years without providing evidence that the examination forms are comparable. Since different test forms were administered across years, the absence of equating procedures, anchor items, or measurement invariance analyses raises concerns about the validity of direct comparisons and the interpretation of trends.

 There are also several inconsistencies and errors in the reporting of results. For example, some tables refer to the year 2025 instead of 2024, and discrepancies exist between values reported in the narrative and those presented in the tables. The reporting of ANOVA statistics is also inconsistent, with degrees of freedom stated differently in the text and tables. Such errors suggest inadequate proofreading and raise concerns about the accuracy of the analyses.

 The discussion section largely repeats the results rather than providing deeper interpretation of the findings. The authors should engage more critically with the literature and explore possible explanations for the observed fluctuations in item difficulty and discrimination, particularly the notable changes observed in 2024. Issues such as curriculum modifications, examination reforms, changes in candidate preparation, and post-pandemic educational effects could be considered. In addition, the literature review would benefit from greater synthesis and engagement with recent psychometric research, particularly studies focusing on longitudinal assessment monitoring and examination quality assurance.

 The manuscript also requires substantial language editing. Numerous grammatical errors, awkward sentence constructions, repetitions, and unclear expressions reduce the readability of the paper. In some sections, sentences appear duplicated or poorly structured, which detracts from the overall quality of the manuscript.

 In conclusion, the study addresses an important topic and has the potential to contribute to the field of educational measurement and assessment. However, the current version contains significant methodological and reporting weaknesses that must be addressed. I therefore recommend major revision. The manuscript may become suitable for publication after the authors carefully address the methodological concerns, correct statistical inconsistencies, strengthen the discussion and literature review, improve reporting transparency, and undertake thorough language editing.

Is the work clearly and accurately presented and does it cite the current literature?

Yes

If applicable, is the statistical analysis and its interpretation appropriate?

No

Are all the source data underlying the results available to ensure full reproducibility?

No

Is the study design appropriate and is the work technically sound?

No

Are the conclusions drawn adequately supported by the results?

Yes

Are sufficient details of methods and analysis provided to allow replication by others?

No

Reviewer Expertise:

Educational Measurment and Evaluation, Bias in Psychometric

I confirm that I have read this submission and believe that I have an appropriate level of expertise to confirm that it is of an acceptable scientific standard, however I have significant reservations, as outlined above.

# Response

**Published:** 2026-06-29

**Authors:** Odunayo Odumbo

**Affiliations:** Humanities, Kampala International University - Western Campus, Bushenyi, Western Region, Uganda

Reviewer comment - **Omale Onuh**

**Reviewer comment: **Despite these strengths, the manuscript requires substantial revision before it can be considered suitable for indexing. Several methodological, statistical, conceptual, and presentation issues need to be addressed. First, there is a mismatch between the stated objectives and the analyses conducted. While distractor efficiency was included among the psychometric characteristics to be examined, no results or analyses relating to distractor efficiency were presented. The authors should either provide the relevant analyses or revise the objectives accordingly.

***Author's Comments:** Thank you for your valuable observation. We agree that there was an inconsistency between the stated objectives and the analyses presented. To address this issue, the study objectives have been revised to align with the psychometric characteristics actually examined in the study. Specifically, references to distractor efficiency have been removed from the objectives, research questions, and related sections, as no distractor efficiency analyses were conducted. This revision ensures consistency between the study aims, methodology, results, and conclusions throughout the revised manuscript.*

**Reviewer comment: **The methodology section lacks sufficient detail to allow replication of the study. Important information regarding the procedures used for item analysis, computation of psychometric indices, handling of missing data, criteria for classifying item difficulty and discrimination levels, and statistical software employed is not adequately described. Furthermore, the study compares psychometric characteristics across different examination years without providing evidence that the examination forms are comparable. Since different test forms were administered across years, the absence of equating procedures, anchor items, or measurement invariance analyses raises concerns about the validity of direct comparisons and the interpretation of trends.

***Author's Comments:** Thank you for your valuable comments. The inconsistency identified in the stated objectives has been corrected in the revised manuscript. In addition, the Methodology section has been expanded to provide a more detailed description of the item analysis procedures, the computation of psychometric indices, the handling of missing data, and the criteria used to classify item difficulty and discrimination levels. The statistical software used for the analyses has also been clearly specified. Furthermore, we have acknowledged and justified the absence of formal test equating, anchor items, and measurement invariance analyses, clarifying that the study was designed as a longitudinal quality monitoring investigation within the Classical Test Theory framework rather than as an equating study.*

**Reviewer comment: **There are also several inconsistencies and errors in the reporting of results. For example, some tables refer to the year 2025 instead of 2024, and discrepancies exist between values reported in the narrative and those presented in the tables. The reporting of ANOVA statistics is also inconsistent, with degrees of freedom stated differently in the text and tables. Such errors suggest inadequate proofreading and raise concerns about the accuracy of the analyses.

***Author's Comments:** Thank you for your valuable observations. The inconsistencies in the reported years were due to coding errors, which have now been corrected. Consequently, the ANOVA results and all related statistical values have been updated accordingly in the revised manuscript to ensure accuracy and consistency.*

**Reviewer comment: **The discussion section largely repeats the results rather than providing deeper interpretation of the findings. The authors should engage more critically with the literature and explore possible explanations for the observed fluctuations in item difficulty and discrimination, particularly the notable changes observed in 2024. Issues such as curriculum modifications, examination reforms, changes in candidate preparation, and post-pandemic educational effects could be considered. In addition, the literature review would benefit from greater synthesis and engagement with recent psychometric research, particularly studies focusing on longitudinal assessment monitoring and quality assurance in examinations.

***Author's Comments:** Thank you for your valuable observation. The Discussion section has been revised to incorporate the potential influence of curriculum modifications, examination reforms, changes in candidate preparation, and post-pandemic educational disruptions on the interpretation of the study findings. These additions provide a broader context for the observed psychometric trends and strengthen the interpretation of the results in the revised manuscript.*

**Reviewer comment:** The manuscript also requires substantial language editing. Numerous grammatical errors, awkward sentence constructions, repetitions, and unclear expressions reduce the readability of the paper. In some sections, sentences appear duplicated or poorly structured, which detracts from the overall quality of the manuscript.

 In conclusion, the study addresses an important topic and has the potential to contribute to the field of educational measurement and assessment. However, the current version contains significant methodological and reporting weaknesses that must be addressed. I therefore recommend major revision. The manuscript may become suitable for publication after the authors carefully address the methodological concerns, correct statistical inconsistencies, strengthen the discussion and literature review, improve reporting transparency, and undertake thorough language editing.

***Author's Comments:** Thank you for your constructive comments. In response, the manuscript has been thoroughly language-edited to enhance its clarity, coherence, grammatical accuracy, and overall readability.*
